# Prognostic factors for gastrectomy in elderly patients with gastric cancer

**DOI:** 10.1186/s12957-017-1131-6

**Published:** 2017-03-11

**Authors:** Daisuke Ueno, Hideo Matsumoto, Hisako Kubota, Masaharu Higashida, Takashi Akiyama, Akiko Shiotani, Toshihiro Hirai

**Affiliations:** 1 Department of Digestive Surgery, Kawasaki Medical School, 577 Matsushima, Kurashiki, Okayama 701-0192 Japan; 2 Department of Pathology, Kawasaki Medical School, 577 Matsushima, Kurashiki, Okayama 701-0192 Japan; 3 Department of Gastroenterology, Kawasaki Medical School, 577 Matsushima, Kurashiki, Okayama 701-0192 Japan

**Keywords:** Gastric cancer, Elderly patient, Prognostic factor, Postoperative complications

## Abstract

**Background:**

The aim of the present study was to investigate the age-specific prognostic factors in patients who underwent gastrectomy for gastric cancer.

**Methods:**

The medical records of 366 patients with gastric cancer who underwent surgical resection at our hospital between January 2007 and December 2014 were retrospectively reviewed. Of the 366 patients, 117 were aged 75 years or older and 249 were aged 74 years or younger. All factors that were identified as significant using univariate analysis were included in the multivariate analysis.

**Results:**

The median follow-up duration was 52.9 months (range, 1.0–117.5 months). We found that in patients aged 75 years or older, postoperative complications and the extent of cancer were independent prognostic factors of overall survival and disease-free survival. In contrast, in patients aged 74 years or younger, only the lymph node status and postoperative chemotherapy were independent prognostic factors for overall survival and disease-free survival, respectively.

**Conclusions:**

Pathological outcomes and postoperative complications are important prognostic factors for survival in patients aged 75 years or older with gastric cancer, whereas pathological outcomes and postoperative chemotherapy are important prognostic factors for survival in patients aged 74 years or younger. Because the prevention of postoperative complications may contribute to improvements in the prognosis of elderly patients with gastric cancer, we suggest that it is necessary to consider limited surgery instead of radical surgery, depending on the patient’s general condition and co-morbidities.

## Background

Gastric cancer is the fourth most common malignant disease and the second leading cause of cancer-related deaths worldwide [1]. In recent years, mortality from gastric cancer has significantly decreased in Japan because of advances in diagnostic and treatment modalities, including improvements in screening, surgery, and chemotherapy [2]. Radical gastrectomy is the mainstay of curative treatment for gastric cancer. The incidence of gastric cancer remains relatively high, and particularly in Japan, the gastric cancer incidence in elderly patients has been increasing along with the increasing life expectancy [3]. Characteristics of elderly patients such as declining physiological function, poor nutritional status, and surgical trauma from radical gastrectomy appear to result in higher postoperative morbidity, prolonged hospital stays, increased healthcare costs, and higher postoperative mortality. Elderly patients might have poorer prognoses compared with younger patients primarily because of the increased risk of postoperative complications. Perioperative nutritional support and preoperative rehabilitation are beneficial for elderly patients with gastric cancer and can reduce surgical complications and mortality [4, 5].

As the elderly population increases, it becomes increasingly more important to understand how best to treat elderly patients with gastric cancer. In the present retrospective study, we aimed to identify the independent prognostic factors for survival in elderly patients (age ≥75 years) with gastric cancer, with a particular focus on preoperative nutritional status and postoperative complications.

## Methods

### Patients

**Fig. 1:**
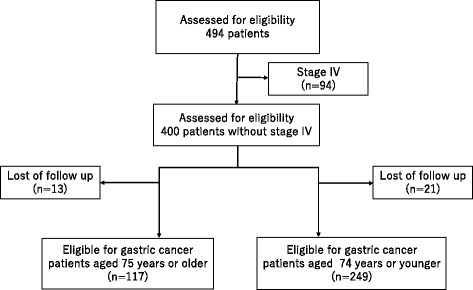
Enrollment and follow-up of the patients

We investigated a total of 494 consecutive patients with a histologically confirmed diagnosis of gastric cancer who were indicated for surgical treatment. They underwent surgery at the Kawasaki Medical School Hospital between January 2007 and December 2014, and their records were reviewed retrospectively. Thirty-four patients were lost to follow-up, and 94 patients who had metastases to other organs were excluded. The remaining 366 patients were enrolled in this study. The patients were divided into two groups according to age: 117 patients aged 75 years or older (group A) and 249 patients aged 74 years or younger (group B) (Fig. 1). Comorbidities were estimated using the Charlson Comorbidity Index (CCI) [6] with the exclusion of gastric cancer as a comorbidity. The pathological characteristics of the tumors were assessed according to the 3rd English edition of the Japanese Classification of Gastric Carcinoma [7]. The activities of daily living were evaluated using the American Society of Anesthesiologists-Physical Status (ASA-PS) scale [8]. We have summarized the patient characteristics in Table 1. The Institutional Review Board of Kawasaki Medical School approved this study (No. 1935).

**Table 1:** Patient characteristics

	*N* = 366
Age	
<30/31–40/41–50/51–60	1/7/11/61
61–70/71–80/81–90/91<	118/120/44/4
Sex (male; female)	267/99
ASA-PS 1/2/3	210/121/35
CCI 2/3/4/5	204/96/37/29
CCI^a^ 0/1/2/3/4/5	183/80/52/26/15/10
Surgery	
DG/TG/LADG/cardiectomy/LR	134/86/105/28/13
Combined resection	
Yes	81
No	285
Pathological stage I/II/III	236/70/60
Postoperative complications	
C. D. grade I/II/III/IV/V	11/17/16/2/3
Curability A/B/C	362/4/0
Onodera PNI <40/≥40	47/319
Chemotherapy	
Present	91
Absent	275

*ASA-PS* American Society of Anesthesiologists-Physical Status, *CCI* Charlson comorbidity score, *DG* distal gastrectomy, *TG* total gastrectomy, *LADG* laparoscopic-assisted distal gastrectomy, *LR* local resection, *C.D*. Clavien–Dindo, *PNI* prognostic nutritional index

^a^Without gastric cancer

### Pathological diagnosis

Histopathologically, papillary and tubular adenocarcinomas were classified as intestinal type adenocarcinomas, and poorly differentiated signet ring cell and mucinous adenocarcinomas were classified as diffuse type adenocarcinomas. The degrees of lymphatic (ly0, 1, and 2) and venous (v0, 1, and 2) invasion were defined according to the Japanese Classification of Gastric Carcinoma [7]. Tumors were classified according to size as ≥5 or <5 cm.

### Inflammatory responses and nutrition

The systemic inflammatory responses were classified into three groups according to the modified Glasgow Prognostic Score (mGPS 0, 1, and 2). The GPS was modified as follows: 0 = serum C-reactive protein (CRP) concentration ≤10 mg/L, 1 = CRP >10 mg/L and serum albumin concentration ≥35 g/L, and 2 = CRP >10 mg/L and albumin <35 g/L [9]. The Onodera prognostic nutritional index (PNI) was determined according to the following formula: 10 × serum albumin concentration (g/dL) + 0.005 × total lymphocyte count (/mm^3^) [10]. The Onodera PNI was implemented in the following scoring system: good nutrition, >50; mild malnutrition, 45–50; moderate malnutrition, 40–45; and severe malnutrition, <40.

### Surgery

The details of the surgical procedures are given in Table 1. Patients who underwent total or distal gastrectomy also underwent D1+ or D2 lymph node dissection in accordance with the Japanese gastric cancer treatment guidelines 2010 (ver. 3) [7]. In distal gastrectomy, the resected nodes included No. 1, 3, 4sd, 4d, 5, 6, 7, 8a, and 9 for D1+ lymph node dissection, and additional nodes No. 11p and 12a for D2 lymph node dissection. In total gastrectomy, the resected nodes included No. 1, 2, 3, 4sa, 4sb, 4d, 5, 6, 7, 8a, 9, and 11p for D1+ lymph node dissection, and additional nodes No. 10, 11d, and 12a for D2 lymph node dissection. In principle, D1+ lymphadenectomy was performed in patients with cT1N0 tumors for whom endoscopic mucosal resection or endoscopic submucosal dissection was indicated and in those who demonstrated signs of metastatic invasion. D2 lymphadenectomy was performed in patients with potentially curable stage T2–4 tumors and in those with cT1NX tumors.

Briefly, as a part of partial cardiectomy, lymph node dissection along the lesser curvature and left pericardial portion of the stomach was performed, followed by the incision of two or three branches of the upper short gastric vessels to create the new fornix and the anastomosis of the primary fornix to the distal edge of the subsequent gastrectomy [11]. Local resection was performed with a 2-cm cancer-free margin. The area for lymphadenectomy was determined by the lymphatic flow from the tumor [12, 13].

### Postoperative complications

Postoperative complications were assessed according to the Clavien–Dindo classification [14]. Surgical site infections (SSIs) were defined in accordance with the criteria of the Center for Disease Control and Prevention [15]. The incisional SSI diagnosis criteria included infections that occurred within 30 days after surgery.

### Follow-up

The follow-up schedule of patients with pT1 tumors after gastrectomy included physical examinations, serum analyses (including analyses of carcinoembryonic antigen, carbohydrate antigen 19-9, and carbohydrate antigen 125), abdominal ultrasonography or computed tomography performed at least once every 6 months, and an annual gastroscopy for a period of 5 years. In patients with pT2–4 tumors, the follow-up schedule after gastrectomy included the examinations described for pT1 tumors; however, they were performed at least once every 3 months for a period of 3 years, and an annual gastroscopy for a period of 5 years.

### Chemotherapy

Tegafur-gimestat-otastat potassium (TS-1) was administered as first-line adjuvant chemotherapy in patients who underwent curative gastrectomy for stage II and III tumors in accordance with the findings and guidelines of the Adjuvant Chemotherapy Trial of TS-1 for Gastric Cancer study [16]. TS-1 plus paclitaxel or cisplatin, or TS-1 monotherapy in cases of high-risk patients, was administered to patients with stage IV disease who had undergone gastrectomy.

### Statistical analyses

Overall survival (OS) was defined as the interval between surgery and the date of death due to any cause. Disease-specific survival (DSS) was defined as the interval between surgery and the date of death from gastric cancer. Patients who were lost to follow-up were excluded. The OS and DSS were calculated using the Kaplan–Meier method and compared using the log-rank test. Univariate and multivariate analyses were performed using the Cox proportional hazards model. *P* values <0.05 were defined as statistically significant. All statistical analyses were performed using JMP 10 software (SAS Institute, Cary, NC, USA).

## Results

The median follow-up duration was 52.9 months (range, 1.0–117.5 months). One patient died within 30 days after surgery. At the end of the follow-up period, 74 patients had died, 41 from unrelated causes including respiratory disease (34.1%), other cancers (19.5%), senile dementia (17.1%), circulatory disease (12.2%), cerebrovascular disease (7.3%), suicide (2.4%), and unclear cause but not cancer (7.3%), respectively.

In group A, combined resection included 1 case of colon resection, 25 cases of gallbladder resection, 1 case of distal pancreatectomy, 4 cases of spleen resection, and 1 case of kidney resection. In group B, combined resection included 7 cases of colon resection, 16 cases of gallbladder resection, 7 cases of distal pancreatectomy, 30 cases of spleen resection, and 1 case of kidney resection. Postoperative complications are summarized in Table 2. The total number of postoperative complications was 20 in group A and 29 in group B. There were no significant differences between type of surgeries. The CCI of group A was significantly higher than that of group B (3.37 vs. 2.52; *p* < 0.001). Similar results were obtained for all other conditions except for gastric cancer (1.84 vs. 0.67; *p* < 0.001). In addition, patients with tumors other than gastric cancer were significantly more common in group A than in group B (28 vs. 19; *p* < 0.001). The 5-year OS rates for stage I, II, III, and IV gastric cancers were 90.7, 72.8, 44.6, and 13.4%, respectively, and the 5-year DSS rates were 99.5, 83.6, 56.6, and 14.5%, respectively.

**Table 2:** Postoperative complications in groups A and B

	Group A (*n* = 117)	Group B (*n* = 249)
Incisional SSI	5 (4.3%)	4 (1.6%)
Pancreatic fistula	3 (2.6%)	6 (2.4%)
Anastomotic leakage	3 (2.6%)	10 (4.0%)
Pneumonia	2 (1.7%)	2 (0.8%)
AKI	2 (1.7%)	0 (0%)
Anastomotic ulcer	1 (0.8%)	1 (0.4%)
Anastomotic edema	1 (0.8%)	0 (0%)
Pulmonary embolism	1 (0.8%)	0 (0%)
Sigmoid colon volvulus	1 (0.8%)	0 (0%)
Sudden death	1 (0.8%)	0 (0%)
CRBSI	0 (0%)	2 (0.8%)
Postoperative bleeding	0 (0%)	2 (0.8%)
Chylous ascites	0 (0%)	2 (0.8%)

*SSI* surgical site infection, *AKI* acute kidney injury, *CRBSI* catheter-related blood stream infection

A univariate Cox regression analysis was performed with the following variables: sex, ASA-PS score, type of surgery, histology, tumor size, depth of invasion (T), lymphatic invasion (ly), venous invasion (v), lymphatic metastasis (N), postoperative complications classified according to the Clavien–Dindo method [14], Onodera PNI [10], mGPS score [9], and postoperative chemotherapy (Tables 3 and 4). In group A, the ASA-PS score, surgery, tumor size, T, ly, v, N, postoperative complications, and Onodera PNI were identified as significant prognostic factors for both OS and DSS. Subsequently, these factors were included in a multivariate Cox regression analysis, which identified postoperative complications as independent prognostic factors for OS. The ASA-PS score, T, v, N, and postoperative complications were also independent prognostic factors for DSS (Table 3).

**Table 3:** Univariate and multivariate analyses of prognostic factors for overall survival and disease-specific survival in patients aged 75 years or older (*n* = 117)

		Overall survival					Disease-specific survival				
			Univariate analysis		Multivariate analysis			Univariate analysis		Multivariate analysis	
	*n*	MST (months)	Hazard ratio (95% CI)	*P* value	Hazard ratio (95% CI)	*P* value	MST (months)	Hazard ratio (95% CI)	*P* value	Hazard ratio (95% CI)	*P* value
Sex	–	–	–	0.229	–	–	–	–	0.890	–	–
Males	82	74.0	1.16 (0.63–2.24)	–	–	–	–	1.00	–	–	–
Females	35	70.8	1.00	–	–	–	–	1.18 (0.44–3.68)	–	–	–
ASA-PS	–	–	–	0.041*	–	–	–	–	0.014*	–	0.002*
1	30	–	1.00	–	–	–	–	1.00	–	1.00	–
2	65	80.2	1.29 (0.64–2.71)	–	–	–	–	1.12 (0.33–4.31)	–	0.14 (0.01–1.53)	–
3	22	34.3	2.88 (1.30–6.47)	–	–	–	–	3.58 (1.06–13.87)	–	9.11 (1.20–103.77)	–
Surgery	–	–	–	0.040*	–	0.636	–	–	0.036*	–	0.476
DG	50	70.8	1.00	–	1.00	–	–	1.00	–	1.00	–
TG	23	32.4	1.54 (0.73–3.07)	–	1.35 (0.52–3.32)	–	–	1.72 (0.51–5.18)	–	2.48 (0.20–26.78)	–
LADG	25	–	0.36 (0.08–1.07)	–	0.68 (0.14–2.43)	–	–	1.18e^−9^ (0–0.65)	–	1.33e^−9^ (0–28.12)	–
Cardiectomy	11	33.0	2.54 (1.09–5.44)	–	1.75 (0.62–4.62)	–	50.6	3.46 (1.03–10.56)	–	0.23 (0.02–2.35)	–
LR	8	–	0.87 (0.13–3.00)	–	0.71 (0.10–3.20)	–	–	1.16e^−9^ (5.39e^−16^–7.35e^−130^)	–	5.54e^−10^ (0–24.97)	–
Histology	–	–	–	0.420	–	–	–	–	0.134	–	–
Intestinal	76	74.0	1.00	–	–	–	–	1.00	–	–	–
Diffuse	41	51.6	1.30 (0.72–2.30)	–	–	–	–	2.24 (0.88–5.87)	–	–	–
Tumor size	–	–	–	0.018*	–	0.793		–	0.003*	–	0.358
<5 cm	68	80.2	1.00	–	1.12 (0.46–2.81)	–	–	1.00	–	2.69 (0.31–23.75)	–
≥5 cm	49	50.5	1.93 (1.09–3.47)	–	1.00	–	–	3.68 (1.38–11.49)	–	1.00	–
Depth of invasion (T)	–	–	–	<0.001*	–	0.317	–	–	<0.001*	–	0.005*
T1	57	80.2	1.00	–	1.00	–	–	1.00	–	1.00	–
T2	18	88.8	0.54 (0.15–1.47)	–	0.30 (0.06–1.09)	–	–	1.26e^−8^ (0–15.07)	–	2.86e^−10^ (0–2.92)	–
T3	20	38.4	2.16 (1.01–4.48)	–	0.65 (0.18–2.15)	–	–	20.02 (3.55–374.36)	–	15.44 (1.16–642.77)	–
T4	22	25.2	2.50 (1.22–5.03)	–	0.87 (0.27–2.61)	–	50.5	33.63 (6.43–617.14)	–	10.86 (0.65–432.23)	–
Lymphatic invasion (ly)	–	–	–	0.013*	–	0.328	–	–	0.013*	–	0.578
ly0, ly1	73	88.8	1.00	–	1.00	–	–	1.00	–	1.00	–
ly2, ly3	43	41.3	2.29 (1.29–4.08)	–	1.49 (0.66–3.34)	–	–	3.90 (1.51–11.21)	–	1.79 (0.22–14.91)	–
Venous invasion (v)	–	–	–	0.001*	–	0.944	–	–	<0.001*	–	0.013*
v0, v1	77	80.2	1.00	–	1.00	–	–	1.00	–	1.00	–
v2, v3	40	37.2	2.18 (1.22–3.87)	–	1.02 (0.44–2.26)	–	50.6	11.97 (3.94–51.74)	–	9.38 (6.56–82.05)	–
Lymph node metastasis (N)	–	–	–	<0.001*	–	0.178	–	–	<0.001*	–	0.010*
N0	73	88.8	1.00	–	1.00	–	–	1.00	–	1.00	–
N1	20	41.3	2.91 (1.29–6.58)	–	2.41 (0.84–6.84)	–	51.6	6.30 (1.89–24.18)	–	5.40 (0.67–55.78)	–
N2	13	–	3.19 (1.25–7.71)	–	0.92 (0.18–3.80)	–	–	1.65e^−8^ (0–3.37)	–	8.07e^−11^ (0–1.20)	–
N3	11	17.9	8.82 (4.21–19.22)	–	3.76 (0.86–15.50)	–	25.2	28.24 (7.99–115.80)	–	6.70 (0.34–219.73)	–
Clavien–Dindo	–	–	–	<0.001*	–	<0.001*	–	–	<0.001*	–	<0.001*
0	97	70.8	1.00	–	1.00	–	–	1.00	–	1.00	–
I	5	–	0.86 (0.14–2.84)	–	0.52 (0.07–2.21)	–	–	1.24 (0.06–6.35)	–	0.24 (0.01–3.42)	–
II	6	88.8	0.28 (0.01–1.32)	–	0.82 (0.04–5.79)	–	–	6.15e^−9^ (0–2.07)	–	12.78 (0–5.28e^298^)	–
III	5	37.2	1.81 (0.43–5.05)	–	2.33 (0.44–10.33)	–	–	1.92 (0.10–9.83)	–	1.09 (0.01–104.71)	–
IV	1	7.7	27.25 (1.39–184.77)	–	7.69 (0.35–67.41)	–	7.7	8.73e^16^ (18.30–1.89e^109^)	–	8.31e^18^ (15.71-NC)	–
V	3	1.1	5.21e^10^ (87.65–1.64e^31^)	–	1.08e^11^ (57.71–9.25e^141^)	–	1.1	7.72e^31^ (255.05-NC)	–	5.71e^33^ (296.06–3.28e^306^)	–
Onodera PNI	–	–	–	<0.001*	–	0.056	–	–	0.010*	–	0.698
<40	29	33.0	2.65 (1.47–4.69)	–	2.03 (0.97–4.17)	–	–	2.35 (0.86–5.98)	–	1.42 (0.23–9.56)	–
≥40	88	80.2	1.00	–	1.00	–	–	1.00	–	1.00	–
Modified GPS	–	–	–	0.375	–	–	–	–	0.574	–	–
0	96	70.8	1.00	–	–	–	–	1.00	–	–	–
1	7	–	0.82 (0.13–2.71)	–	–	–	–	1.24 (0.06–6.21)	–	–	–
2	14	34.3	1.54 (0.66–3.15)	–	–	–	–	1.59 (0.36–4.89)	–	–	–
Chemotherapy	–	–	–	0.562	–	–	–	–	0.252	–	–
Yes	24	50.6	1.00	–	–	–	–	1.00	–	–	–
No	93	74.0	1.42 (0.74–2.60)	–	–	–	–	2.77 (1.05–7.03)	–	–	–

*DG* distal gastrectomy, *TG* total gastrectomy, *LADG* laparoscopic-assisted distal gastrectomy, *LR* local resection, *PNI* prognostic nutritional index, *GPS* Glasgow prognostic score, *ASA-PS* American Society of Anesthesiologists-Physical Status

*P* values indicated by (*) are statistically significant (*P* < 0.05) NC means “not calculated”

**Table 4:** Univariate and multivariate analyses of prognostic factors for overall survival and disease-specific survival in patients aged 74 years or younger (*n* = 249)

		Overall survival					Disease-specific survival				
			Univariate analysis		Multivariate analysis			Univariate analysis		Multivariate analysis	
	*n*	MST (months)	Hazard ratio (95% CI)	*p* value	Hazard ratio (95% CI)	*p* value	MST (months)	Hazard ratio (95% CI)	*p* value	Hazard ratio (95% CI)	*p* value
Sex	–	–	–	0.204	–	–	–	–	0.246	–	–
Males	185	–	1.96 (0.75–6.72)	–	–	–	–	2.35 (0.65–15.04)	–	–	–
Females	64	–	1.00	–	–	–	–	1.00	–	–	–
ASA-PS	–	–	–	<0.001*	–	0.274	–	–	0.777	–	–
1	180	–	1.00	–	1.00	–	–	1.00	–	–	–
2	56	–	2.37 (0.90–5.69)	–	2.17 (0.09–6.14)	–	–	0.74 (0.11–2.72)	–	–	–
3	13	76.4	6.62 (1.86–18.61)	–	3.23 (0.50–16.85)	–	–	1.74 (0.09–8.97)	–	–	–
Surgery	–	–	–	0.023*	–	0.932	–	–	0.023*	–	0.935
DG	84	–	1.00	–	1.00	–	–	1.00	–	1.00	–
TG	63	96.9	1.29 (0.55–2.96))	–	1.20 (0.44–3.28)	–	–	0.76 (0.24–2.15)	–	1.95 (0.3–11.86)	–
LADG	80	–	0.17 (0.03–0.61)	–	0.69 (0.09–3.72)	–	–	2.67e^−10^ (0–0.23)	–	1.75e^−8^ (0–83.40)	–
Cardiectomy	15	–	0.33 (0.01–1.66)	–	1.19 (0.06–9.75)	–	–	2.67e^−10^ (0–1.00)	–	(0-NC)	–
LR	7	–	3.17e^−9^ (1.12e^−55^–2.10)	–	7.82e^−9^ (4.43e^−54^–13.53)	–	–	2.67e^−10^ (9.69e^−301^–2.97)	–	0.20 (4.22e^−59^-NC)	–
Histology	–	–	–	0.205	–	–	–	–	0.066	–	–
Intestinal	110	–	1.00	–	–	–	–	1.00	–	–	–
Diffuse	139	–	1.70 (0.76–4.15)	–	–	–	–	3.08 (0.98–13.52)	–	–	–
Tumor size (cm)	–	–	–	0.002*	–	0.637	–	–	<0.001*	–	0.810
<5 cm	172	–	1.00	–	1.00	–	–	1.00	–	1.00	–
≥5 cm	77	–	3.19 (1.47–7.03)	–	1.32 (0.41–4.11)	–	–	10.63 (3.37–46.69)	–	0.76 (0.08–8.99)	–
Depth of invasion (T)	–	–	–	<0.001*	–	0.172	–	–	<0.001*	–	0.013*
T1	154	–	1.00	–	1.00	–	–	1.00	–	1.00	–
T2	33	–	2.29 (0.48–8.69)	–	0.64 (0.08–4.25)	–	–	1.75e^9^ (0.80–9.06e^145^)	–	1.22e^9^ (0.01–2.39e^139^)	–
T3	23	–	4.04 (0.85–15.42)	–	1.27 (0.18–8.21)	–	–	2.70e^9^ (1.24–1.54e^260^)	–	5.48e^9^ (0.03–3.32e^96^)	–
T4	39	–	13.16 (5.23–37.50)	–	3.02 (0.65–17.33)	–	–	2.41e^10^ (31.03–1.23e^144^)	–	6.14e^10^ (0.89–1.81e^81^)	–
Lymphatic invasion (ly)	–	–	–	0.045*	–	0.189	–	–	0.003*	–	0.009*
ly0, ly1	192	–	1.00	–	1.00	–	–	1.00	–	1.00	–
ly2, ly3	57	–	2.94 (1.31–6.41)	–	0.37 (0.07–1.60)	–	–	4.20 (1.50–12.00)	–	0.05 (0.01–0.51)	–
Venous invasion (v)	–	–	–	0.005*	–	0.815	–	–	0.029*	–	0.271
v0, v1	198	–	1.00	–	1.00	–	–	1.00	–	1.00	–
v2, v3	51	–	2.95 (1.29–6.43)	–	0.87 (0.26–2.88)	–	–	2.99 (1.00–8.29)	–	0.40 (0.07–2.02)	–
Lymph node metastasis (N)	–	–	–	<0.001*	–	0.008*	–	–	<0.001*	–	<0.001*
N0	180	–	1.00	–	1.00	–	–	1.00	–	1.00	–
N1	30	–	4.24 (1.42–11.76)	–	1.96 (0.41–8.34)	–	–	24.94 (3.69–487.50)	–	27.56 (1.97–1080.37)	–
N2	20	–	2.66 (0.41–10.38)	–	0.94 (0.11–5.91)	–	–	21.84 (2.09–470.13)	–	13.89 (0.94–453.66)	–
N3	19	66.1	14.93 (5.71–39.31)	–	10.55 (1.99-73.65)	–	66.1	97.33 (17.79–807.31)	–	208.72 (10.54–14986.60)	–
Clavien–Dindo	–	–	–	0.654	–	–	–	–	0.235	–	–
0	220	–	1.00	–	–	–	–	1.00	–	–	–
I	6	–	2.09 (0.12–10.05)	–	–	–	–	3.65 (0.20–18.82)	–	–	–
II	11	–	2.09 (0.33–7.15)	–	–	–	–	1.91 (0.10–9.82)	–	–	–
III	11	–	2.18 (0.35–7.46)	–	–	–	–	4.27 (0.66–15.93)	–	–	–
IV	1	–	1.17e^−7^ (0–86.79)	–	–	–	–	2.27e^−7^ (0–143.14)	–	–	–
Onodera PNI	–	–	–	<0.001*	–	0.091	–	–	0.008*	–	0.261
<40	18	86.8	8.25 (3.17–19.21)	–	4.92 (0.76–25.22)	–	–	4.75 (1.07–15.12)	–	6.47 (0.22–158.87)	–
≥40	231	–	1.00	–	1.00	–	–	1.00	–	1.00	–
Modified GPS	–	–	–	<0.001*	–	0.328	–	–	<0.001*	–	0.865
0	227	–	1.00	–	1.00	–	–	1.00	–	1.00	–
1	10	–	2.98 (0.47–10.34)	–	3.61 (0.49–16.92)	–	–	2.44 (0.13–12.55)	–	1.31 (0.05-14.33)	–
2	12	36.3	15.12 (5.34–37.46)	–	2.20 (0.25–14.83)	–	–	10.52 (2.34–34.66)	–	2.32 (0.04–35.52)	–
Chemotherapy	–		–	<0.001*	–	0.196	–	–	<0.001*	–	0.038*
Yes	67	–	1.00	–	1.00	–	–	1.00	–	1.00	–
No	182	–	5.87 (2.68–13.49)	–	2.84 (0.57–13.55)	–	–	45.67 (9.16–827.80)	–	43.58 (1.20-3226.69)	–

*DG* distal gastrectomy, *TG* total gastrectomy, *LADG* laparoscopic-assisted distal gastrectomy, *LR* local resection, *PNI* prognostic nutritional index, *GPS* Glasgow prognostic score, *ASA-PS* American Society of Anesthesiologists-Physical Status

*p* values indicated by (*) are statistically significant (*p* < 0.05) NC means “not calculated”

The same factors were included in a univariate Cox regression analysis for group B, which identified the ASA-PS score, surgery, tumor size, T, ly, v, N, Onodera PNI, mGPS score, and chemotherapy as independent prognostic factors for OS. In addition, surgery, tumor size, T, ly, v, N, Onodera PNI, mGPS score, and chemotherapy were identified as independent prognostic factors for DSS. Subsequently, these factors were included in a multivariate Cox regression analysis, which identified N as an independent prognostic factor for OS. T, ly, N, and chemotherapy were identified as independent prognostic factors for DSS (Table 4). These findings of the univariate and multivariate analyses are summarized in Tables 5 and 6.

**Table 5:** Significant prognostic factors for overall survival and disease-specific survival in patients older than 75 years

Overall survival		Disease-specific survival	
Univariate analysis	Multivariate analysis	Univariate analysis	Multivariate analysis
ASA-PS	Postoperative complications	ASA-PS	ASA-PS
Surgery		Surgery	T
Tumor size		Tumor size	v
T		T	N
ly		ly	Postoperative complications
v		v	
N		N	
Postoperative complications		Postoperative complications	
Onodera PNI		Onodera PNI	

*ASA-PS* American Society of Anesthesiologists-Physical Status, *PNI* prognostic nutritional index

**Table 6:** Significant prognostic factors for overall survival and disease-specific survival in patients younger than 74 years

Overall survival		Disease-specific survival	
Univariate analysis	Multivariate analysis	Univariate analysis	Multivariate analysis
ASA-PS	N	Surgery	T
Surgery		Tumor size	ly
Tumor size		T	N
T		ly	Chemotherapy
ly		v	
v		N	
N		Onodera PNI	
Onodera PNI		Modified GPS	
Modified GPS		Postoperative chemotherapy	
Postoperative chemotherapy			

*ASA-PS* American Society of Anesthesiologists-Physical Status, *GPS* Glasgow prognostic score, *PNI* prognostic nutritional index

## Discussion

Surgical resection is the main curative treatment option for patients with gastric cancer. However, disease recurrence occurs in 20–60% of these patients despite curative resection [17–19]. The high mortality and relapse rates in patients with gastric cancer are attributable to the extent of surgery and aggressiveness of the cancer [20, 21].

The potential benefits of surgery for elderly patients with gastric cancer must be explored in the context of their shorter life expectancy compared to younger patients. Moreover, elderly patients are more likely to exhibit functional declines in some organs, making it more difficult to overcome surgical stress. We hypothesized that postoperative complications and preoperative nutritional status might be risk factors after gastrectomy for gastric cancer, particularly in patients aged 75 years or older.

The development of postoperative complications is an important prognostic indicator in patients with cancer, and technical complications such as anastomotic leakage, vocal cord paralysis, and chylothorax have a major negative effect on survival after esophagogastrectomy [22]. Indeed, anastomotic leakage has been shown to be a major independent prognostic factor in all patients after undergoing gastrectomy for cancer [23] and a major independent prognostic factor for long-term survival in patients with advanced gastric cancer [24, 25]. The development of postoperative complications has also been reported to be an independent prognostic factor for long-term survival after surgery for esophageal cancer [24, 26, 27].

Several studies have reported the importance of nutritional support during the perioperative period. Enteral nutrition via a nasoenteral feeding tube during the early postoperative period is associated with significantly fewer postoperative complications in patients who undergo radical surgery for gastric cancer [28]. In addition, it was reported that patients with severe malnutrition and gastrointestinal dysfunction might benefit from preoperative parenteral nutrition [29]. Enteral plus parenteral nutrition is the optimal means of administering postoperative nutritional support in elderly patients with gastric cancer [30].

The incorporation of the Onodera PNI into a nutritional scoring system is a simple and useful method for identifying patients at an increased risk of postoperative complications and for predicting long-term survival after total gastrectomy [31]. This index serves as a valuable preoperative clinical marker and a prognostic indicator in elderly patients with colorectal cancer [32].

Hirai et al. [31] reported that surgical stress and postoperative complications had adverse effects on the prognoses of patients undergoing cancer surgery. This phenomenon is referred to as “surgical oncotaxis”, and it has become increasingly recognized as an important factor in multidisciplinary cancer treatments. Minimal invasiveness is therefore an important principle of cancer surgery.

In the present study, we demonstrated that the stage of cancer progression, Onodera PNI, and postoperative complications were prognostic indicators for OS and DSS in patients with gastric cancer aged 75 years or older. Moreover, a multivariate analysis identified postoperative complications as independent prognostic factors of survival and the Onodera PNI was associated with the incidence of postoperative complications in older patients. In contrast, postoperative chemotherapy was the only independent prognostic factor for DSS other than the extent of the tumor in patients aged 74 years or younger. Our results suggest that postoperative complications might be more deleterious in elderly patients than in younger patients. Elderly patients might experience more severe complications owing to the characteristics of surgery, such as severe invasiveness. Therefore, nutrition and rehabilitation must be provided to elderly patients after surgery, less invasive surgical methods should be selected, and postoperative complications should be prevented as much as possible.

As described above, the CCI of group A was significantly higher than in group B. Moreover, patients whose CCI score was 5 were significantly more likely to be in group A (18 vs. 11; *p* < 0.001). Frenkel et al. showed that patients whose CCI score was 5 or more had higher rates of 3-month (odds ratio (OR) = 3.6), 1-year (OR = 7.1), and 5-year (OR = 52.4) mortality than those with a CCI score of 0 [33].

Many postoperative complications result in infections. The precise mechanism of postoperative complications influencing the prognosis remains to be determined. However, there are two possible mechanisms [34]: (1) The enhancement of innate biological factors during infection or those produced by the infection-causing bacteria may directly activate cancer cells to proliferate and acquire metastatic potential. Cytokines such as tumor necrosis factor-α, interleukin-1, IL-6, and IL-18; oxygen free radicals; and nitrogen-based biological effectors have all been implicated in promoting cancer cell growth. (2) A deregulated host immune response during infection may also contribute to tumorigenesis. Therefore, postoperative complications could lead to tumor growth, which leads to poorer outcomes. Rausei et al. suggested that D1 lymphadenectomy should be considered in elderly patients and/or patients with highly co-morbid gastric cancer due to their high postoperative complication rates and no significant improvement of their overall survival [35].

In patients with high-risk gastric cancer (e.g., severe aortic valve stenosis, chronic obstructive pulmonary disease, and age >90 years), local resection is used for treating cases with up to submucosal invasion in our department. In addition, all cases of local resection were intended to be curative. Moreover, postoperative complications from local resection were rare in our study (1/15). Only one case had a confirmed recurrence, and all patients survived (mean follow-up 48.0 months).

Elderly patients in our cohort had significantly more comorbidities than younger patients did; these comorbidities might increase the risks of surgery and affect their prognosis. Based on this hypothesis, we usually perform limited surgery for elderly patients instead of curative aggressive surgery. We believed that the present study might prove this hypothesis to be correct. The results of the present study suggest that it is necessary to consider less invasive surgery to reduce postoperative complications as well as radical surgery for the elderly depending on each patient’s general condition.

However, the present study had some limitations including a relatively small sample size, short follow-up duration, and selection bias. We cannot be sure there was no selection bias. However, if radical surgery was possible, surgery was performed regardless of age. Therefore, we think that the risk of selection bias is not large. In addition, the preliminary findings of the present study should be validated in a larger patient cohort with a longer follow-up duration. Nonetheless, our findings have demonstrated the importance of nutritional status and perioperative nutritional support in elderly patients with gastric cancer who undergo gastrectomy.

## Conclusions

Our findings suggest that pathological outcomes and postoperative complications are predictors of survival in patients with gastric cancer aged 75 years or older, whereas pathological outcomes and chemotherapy are predictors of survival in patients aged 74 years or younger. Because the prevention of postoperative complications may contribute to improved prognosis for elderly patients with gastric cancer, we suggest that it is necessary to consider limited surgery instead of radical surgery depending on each patient’s general condition and comorbidities. Further evaluation of potential prognostic factors is imperative for the improvement of long-term outcomes of patients with gastric cancer.
